# Development of a Fundus Image-Based Deep Learning Diagnostic Tool for Various Retinal Diseases

**DOI:** 10.3390/jpm11050321

**Published:** 2021-04-21

**Authors:** Kyoung Min Kim, Tae-Young Heo, Aesul Kim, Joohee Kim, Kyu Jin Han, Jaesuk Yun, Jung Kee Min

**Affiliations:** 1 Data Scientist Team, BEGAS Inc., Sejong-daero 39, Jung-gu, Seoul 04513, Korea; ganaaml2002@naver.com; 2 Department of Information and Statistics, Chungbuk National University, Chungdae-ro 1, Seowon-gu, Cheongju-si, Chungbuk 28644, Korea; theo@cbnu.ac.kr; 3 Laboratory of Pharmacology, College of Pharmacy and Medical Research Center, Chungbuk National University, 194-31 Osongsaengmyeong 1-ro, Osong-eup, Heungdeok-gu, Cheongju-si, Chungbuk 28160, Korea; aeseulkim@daum.net (A.K.); joohyee4u@naver.com (J.K.); 4 Department of Ophthalmology, Ulsan University Hospital, College of Medicine, University of Ulsan, 877, Bangeojinsunhwando-ro, Dong-gu, Ulsan 44033, Korea; hankj229@hanmail.net

**Keywords:** artificial intelligence, class activation map, convolutional neural network, fundus photograph, retinal diseases

## Abstract

Artificial intelligence (AI)-based diagnostic tools have been accepted in ophthalmology. The use of retinal images, such as fundus photographs, is a promising approach for the development of AI-based diagnostic platforms. Retinal pathologies usually occur in a broad spectrum of eye diseases, including neovascular or dry age-related macular degeneration, epiretinal membrane, rhegmatogenous retinal detachment, retinitis pigmentosa, macular hole, retinal vein occlusions, and diabetic retinopathy. Here, we report a fundus image-based AI model for differential diagnosis of retinal diseases. We classified retinal images with three convolutional neural network models: ResNet50, VGG19, and Inception v3. Furthermore, the performance of several dense (fully connected) layers was compared. The prediction accuracy for diagnosis of nine classes of eight retinal diseases and normal control was 87.42% in the ResNet50 model, which added a dense layer with 128 nodes. Furthermore, our AI tool augments ophthalmologist’s performance in the diagnosis of retinal disease. These results suggested that the fundus image-based AI tool is applicable for the medical diagnosis process of retinal diseases.

## 1. Introduction

Since the Food and Drug Administration’s approval of the IDx-DR system for diabetic retinopathy diagnosis [1], deep learning-based tools have been extensively developed in ophthalmology. Various retinal diseases have useful features for the development of image-based diagnostic systems. For example, drusen or choroidal neovascularization (CNV), subretinal hemorrhage, and vascular leakage are the pathophysiology of dry or neovascular age-related macular degeneration (dAMD or nAMD). These pathologies represent potential photographic sources for the development of artificial intelligence (AI) diagnostic tools [2]. Moreover, other retinal diseases also have diagnostic markers, allowing ophthalmologists to identify retinal changes and differentiates conditions with peer review [3]. Diabetic retinopathy (DR) is characterized by microaneurysms, capillary nonperfusion, and ischemia within the retina [4,5]. In DR, block of blood vessels and irregular in diameter [6] associated with fluid leakage and hemorrhage, which may induce vision loss. In addition, neovascularization is a pathological process in DR [7]. The epiretinal membrane (ERM) is an epimacular membranous tissue, which develops in relation to proliferative disease, inflammation, uveitis, or trauma, and other causes. ERM can lead to tangential traction, causing retinal changes, such as retinal layer thickening, surface wrinkling, and/or nerve fiber layer fibrillation, which cause decreased visual acuity (VA) and metamorphopsia [8,9,10,11,12]. Rhegmatogenous retinal detachment (RRD) is a vision-threatening disease caused by retinal break, which allows vitreous fluid to escape into the subretinal space. As a result, the sensory retina detaches from the retinal pigment epithelium (RPE) [13]. Retinitis pigmentosa (RP) is an inherited retinal disease characterized by progressive loss of photoreceptors and eventual visual loss [14,15]. The clinical diagnosis of RP is based on the presence of nyctalopia, visual field constriction, bone spicule pigmentation, and reduction in electroretinogram (ERG) amplitude. Macular hole (MH) is a round full-thickness opening in the foveal center. In most cases, it is idiopathic, that is, due to abnormal vitreofoveal traction. The advent of optical coherence tomography (OCT), which showed the partially detached posterior hyaloid, enhanced the understanding of MH formation [16,17]. Retinal vein occlusion (RVO) is a heterogeneous group of disorders that commonly have impaired venous return from the retinal circulation. If the occlusion occurs within or posterior to the optic nerve head, it is labeled central RVO (CRVO), and any obstruction within a tributary is a branch RVO (BRVO). Retinal images with CRVO show the features of venous tortuosity, scattered flame-shaped retinal hemorrhages, and/or cotton wool spots in all four quadrants. Patients with BRVO present with a wedge-shaped pattern of intraretinal hemorrhage in the area drained by the occluded vein [18,19].

The characteristic features of such various retinal diseases can be confirmed in fundus photographs. Therefore, we demonstrated the viability of fundus imaging for the application of a deep learning-based AI diagnostic tool and tested the performance of the developed AI tool for differentially diagnosing retinal diseases, showing the abovementioned characteristic fundus features. Furthermore, we compared the diagnostic performance of ophthalmology residents who used or did not use the AI tool.

## 2. Materials and Methods

### 2.1. Subjects

To select the study groups of patients with retinal diseases, the medical records of patients aged >20 years who had been diagnosed with eight retinal diseases (dAMD, nAMD, DR, ERM, RRD, RP, MH, and RVO) between 1 January 2015, and 30 June 2020, at the Department of Ophthalmology of Ulsan University Hospital, Ulsan, Republic of Korea, were retrospectively reviewed. All subjects (628 eyes) underwent a complete ophthalmic examination that included the best-corrected VA assessment, noncontact tonometry (CT-1P; Topcon Corporation, Tokyo, Japan), fundus examination using slit-lamp ophthalmoscopy, fundus photography (TRC-NW8, Topcon Corporation, Tokyo, Japan), and swept-source OCT (DRI OCT-1 Atlantis or Triton; Topcon Corporation, Tokyo, Japan), and/or fluorescein angiography (FA) and indocyanine green angiography (ICGA) (Heidelberg Retina Angiograph Spectralis; Heidelberg Engineering, Heidelberg, Germany). nAMD (79 eyes) was diagnosed by fundus examination, including one or more features, such as subretinal and/or intraretinal fluid, subretinal hemorrhage, and retinal pigmented epithelial detachment and intraretinal exudate in the macular area using fundus photography and OCT. CNV or polypoidal vascular lesions were detected via FA and ICGA. Based on the category of AMD in age-related eye disease [20], drusen types corresponding to categories 2 and 3 were defined as dAMD (58 eyes). DR (95 eyes) was diagnosed based on the medical history and clinical retinal examination. We confirmed several morphological features of DR, including microaneurysms, intraretinal microvascular abnormalities, and neovascularization, by fundus photography and FA. In ERM (99 eyes), the retinal changes were shown as irregular wrinkling, retinal layer thickening and dragging, and ectopic fovea by fundus photography and OCT. RRD (80 eyes) was diagnosed by fundus examination, showing separation between the neurosensory retina and RPE, with an accumulation of liquefied vitreous in the subretinal space through the retinal breaks. We selected only fundus photographs to confirm these features in this study. RP (50 eyes) was diagnosed based on the presence of nyctalopia, visual field constriction, specific fundus appearance (bone spicule pigmentation, attenuated retinal vessels, mottling and granularity of RPE, and optic nerve head pallor), and reduction in ERG amplitude. We selected only fundus photographs to confirm specific fundus appearance in this study. MH (49 eyes) was diagnosed by fundus photography and OCT, showing full-thickness retinal opening in the foveal center of various sizes with or without posterior hyaloid (stage 3 and 4). RVO (39 eyes) was diagnosed based on the features of venous tortuosity, scattered or wedge flame-shaped retinal hemorrhages, and/or cotton wool spots using fundus photography. In selecting the normal controls (79 eyes), the medical records of patients who visited the ophthalmology outpatient clinic were also reviewed.

The subjects’ demographic distribution of each class is shown in Table 1. When analyzing the difference between groups according to age using the one-way analysis of variance test, DR, RRD, RP, and Control are one homogenous subset, and the remaining groups (dAMD, nAMD, ERM, MH, and RVO) consisted of the other homogenous subsets. In the control group, the average age was 56.7, which is close to the average age of all subjects, 61.2 years. In addition, when the gender distribution between each class was confirmed by Pearson’s chi-square test, there was no significant difference in gender distribution between each group (*p* = 0.775).

### 2.2. Fundus Imaging

Fundus photography (TRC-NW8 and DRI Triton) provides high-quality 16.2-megapixel images, with a 45° field of central macular view. All retinal images were reviewed by a retinal specialist (JKM) to ensure that the photographs had sufficiently high quality to adequately visualize the retina. Those with only one disease on one fundus photograph was selected.

### 2.3. Augmentation of Data

To prevent overfitting of AI models and increase the diversity of the dataset, we applied Keras ImageDataGenerator as the data augmentation method. During training, each image was augmented through several methods. Table 2 shows the values of each arguments used in Keras ImageDataGenerator. The value of argument means the range from 0 to the value. (1) Images were cropped randomly on width for approximately 40% of their size, (2) images were cropped randomly on height for approximately 20% of their size, (3) images were zoomed randomly between 0% and 10%, (4) images were rotated randomly between 1° and 90°, (5) images were randomly flipped or mirrored, and (6) images also were randomly sheared between 0% and 30%. By performing augmentation through the above various methods, the features of each disease were trained at various positions and angles during model training.

### 2.4. Preprocessing

Original fundus images have two types of resolution, 2576 × 1934 pixel and 4496 × 3000 pixels with a 24-bit RGB channel. Each image was cropped to most of the circular part of the fundus and cropped to remove the black border. Then, each cropped image was resized to 512 × 512 pixels as input images for the AI models. Keras ImageDataGenerator (https://keras.io/ (accessed on 29 October 2020)) was used for the augmentation of preprocessed images during training.

### 2.5. Convolutional Neural Network (CNN) Modeling

**Figure 1:**
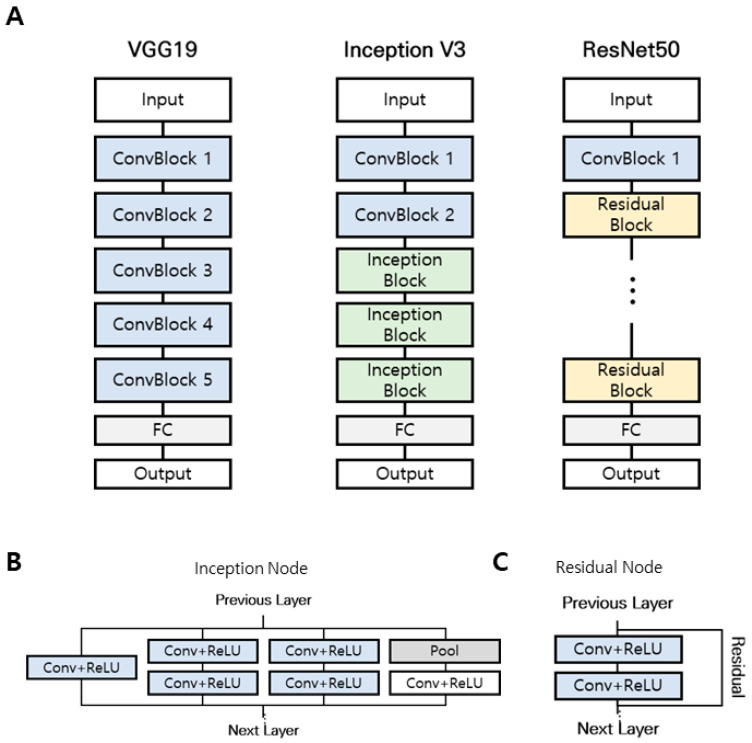
Comparison of three different convolutional neural network (CNN) architectures. (**A**) VGG19 model, Inception V3 model, and ResNet50 model. (**B**) Inception node uses various convolutional filters. (**C**) The residual node maps the necessary information using residual connection.

Three CNN models for training were evaluated (Figure 1), Visual Geometry Group with 19 layers (VGG19) [21], GoogLeNet Inception v3 [22], and Deep Residual Learning for Image Recognition with 50 layers (ResNet50) [23], because these CNN models have been widely applied to various medical image classification tasks. These models are all similar in that they consist of convolutional and pooling layers arranged in sequential units and a final, fully connected layer (Figure 1A). A notable difference among the models is the use of nodes to perform additional operations. The Inception v3 model uses an inception node that reduces the amount of computation while using various convolutional filters (Figure 1B). The ResNet50 model uses a residual node that maps only information that needs to be additionally trained in the layer by connecting information trained in the previous layer to the current layer during training (Figure 1C). We loaded these models trained with image datasets from ImageNet (Accessed 29 October 2020. http://www.image-net.org/) and trained the convolutional layers and fully connected layers with our fundus images. The dataset of macular image was randomly classified into five folds so that 5-fold cross-validation could be performed to estimate model performance. Training was performed using multiple iterations with batch size of 4, learning rate of 0.00001, and Adam optimization. This process was repeated for each model (VGG19, Inception v3, ResNet50) and cross-validation fold.

### 2.6. Cross-Validation of AI-Based Diagnosis

Cross-validation is a useful technique in evaluating the performance of deep learning models. In cross-validation, the dataset was randomly divided into training and test sets, with the training set used to build a model and the test set used to assess the performance of the model by measuring accuracy.

In k-fold cross-validation, the dataset was randomly divided into k subsets of equal size, with one used as a test set and the others as training sets. Cross-validation was performed k times to allow the use of all subsets exactly once as a test set. Model performance was determined according to the average model evaluation scores calculated across the k test subsets. Here, we evaluated the performance of the proposed CNN model using 5-fold cross-validation, with performance determined according to the average accuracy of five cross-validations for each class comparison [2].

### 2.7. Classification Performance Evaluation Index

For diagnosis and accuracy calculation, it was necessary to categorize the model outputs, which are continuous scores. In one-class (normal vs. disease), since the output has a score between 0 and 1, if it is less than 0.5, it was diagnosed as 0 (normal), and if it is greater than or equal to 0.5, it was diagnosed as 1 (disease). In nine-class diagnosis, the class with the highest probability was selected.

Accuracy, sensitivity, specificity, positive predictive value (PPV), and negative predictive value (NPV) were obtained as evaluation indices. Accuracy was defined as the proportion of correctly classified observations. Sensitivity was defined as the ratio of the total number of correctly classified positive examples divided to the total number of positive examples, whereas specificity was defined as the ratio of the total number of correctly classified negative examples divided to the total number of negative examples. PPV was defined as the proportion of predicted positives, which were actual positives, whereas NPV was defined as the proportion of predicted negative, which were actual negatives. The evaluation index was calculated for each class, corresponding to one class and all other classes. The formulas of each evaluation index were expressed as:

$$\mathrm{Accuracy}=\frac{\mathrm{TP}+\mathrm{TN}}{\mathrm{TP}+\mathrm{TN}+\mathrm{FP}+\mathrm{FN}}$$

$$\mathrm{Sensitivity}=\frac{\mathrm{TP}}{\mathrm{TP}+\mathrm{FN}}$$

$$\mathrm{Specificity}=\frac{\mathrm{TN}}{\mathrm{TN}+\mathrm{FP}}$$

$$\mathrm{PPV}\frac{\mathrm{TP}}{\mathrm{TP}+\mathrm{FP}}$$

$$\mathrm{NPV}=\frac{\mathrm{FP}}{\mathrm{TP}+\mathrm{FP}}$$

where TP (true positive) is the number of samples correctly classified as positive, FN (false negative) is the number of samples incorrectly classified as negative, FP (false positive) is the number of samples incorrectly classified as positive, and TN (true negative) is the number of samples correctly classified as negative.

## 3. Results

### 3.1. Two-Class Diagnosis

We achieved a high accuracy for the two-class diagnosis between the normal condition and retinal diseases using 5-fold cross-validation. VGG19 CNN models showed the highest accuracy of 99.12% for two-class diagnosis (normal vs. disease, Table 3). These results suggest that our CNN models are suitable for the preliminary diagnosis process in ophthalmology.

### 3.2. Nine-Class Diagnosis and Visualization

Similar to two-class diagnosis, we performed nine-class diagnosis among the normal condition and eight other diseases using 5-fold cross-validation. Table 4 shows the combination performance of each CNN model and dense layer using 5-fold cross-validation. Each value shows the average test accuracy of all folds plus/minus standard deviation. Unlike two-class diagnostic results, ResNet50 CNN model with 128 nodes showed the highest accuracy of 87.42% for nine-class diagnosis.

For nine-class diagnosis, we finally selected and employed ResNet50 with 128 nodes (Table 5). Our AI tool showed 87.42% accuracy for the diagnosis among normal condition and various retinal diseases (nAMD, dAMD, ERM, RRD, RP, MH, RVO, and DR).

**Figure 2:**
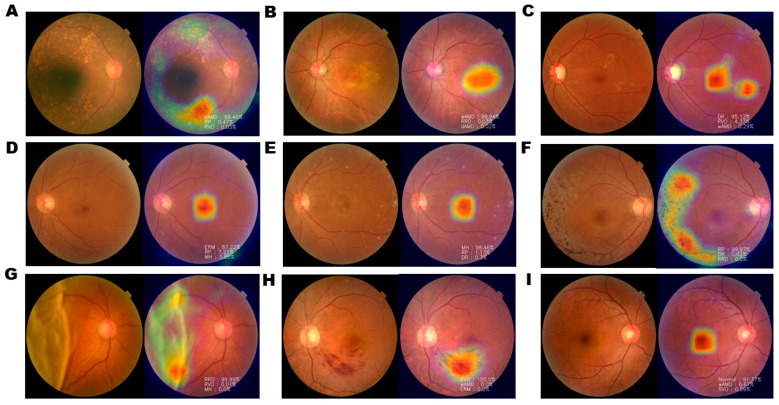
Examples of Gradient-weighted Class Activation Mapping (Grad-CAM) visualization of retinal diseases. Grad-CAM visualization of (**A**) dAMD, (**B**) wAMD, (**C**) DR, (**D**) ERM, (**E**) MH, (**F**) RP, (**G**) RRD, (**H**) RVO, and (**I**) normal retina. Grad-CAM extracts the feature map of the last convolution layer and shows a heatmap within the image describing the calculated weight of the feature map. Heatmap images of nAMD show that the AI tool identified pathological changes, such as drusen, bleeding, elevation of the center, pigmentation, surface wrinkling, and retinal detachment. However, in normal controls, the center of macula is identified, with no degenerated area.

Gradient-weighted Class Activation Map (Grad-CAM) visualization was performed to identify areas showing the greatest effect of retinal diseases. Grad-CAM extracts feature maps of the final convolutional layer of each model trained using fundus images and computes the weights of the feature maps to represent the heatmap in the image (Figure 2).

### 3.3. Classification Probability

**Figure 3:**
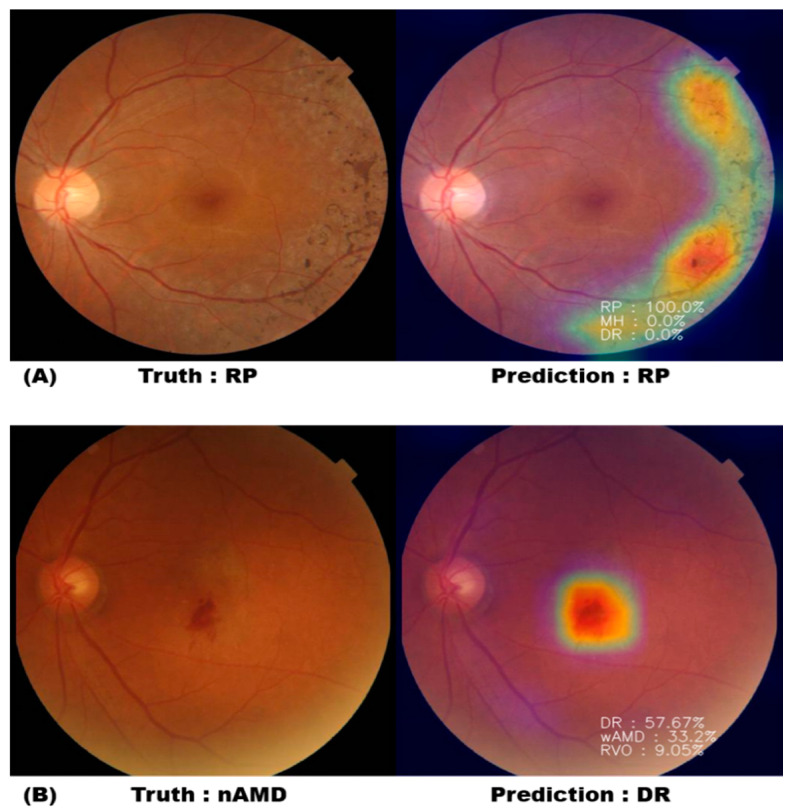
Heatmap and probability with correct classification and misclassification. (**A**) AI correctly diagnosed the RP fundus photograph as RP with 100% probability. (**B**) AI diagnosed the nAMD fundus photograph with 57.67% of DR and 33.2% of nAMD.

In the case of correct classification, when the fundus image with RP characteristics was classified using the trained model, it was classified as RP with 100% probability, and it was shown that symptoms were appropriately identified through the heatmap (Figure 3A). In the case of misclassification, nAMD was diagnosed as DR with 57.67%, nAMD with 33.20%, and RVO with 9.05%, indicating that it was incorrectly diagnosed as DR (Figure 3B). However, it is possible to augment the diagnosis of ophthalmologists by referring to the identification area and proposed disease probability through the heatmap provided by our AI tool.

### 3.4. Comparison of Accuracy Values of the Deep Learning Diagnostic Tool and Residents in Ophthalmology

To compare the performance between AI diagnosis and clinical reviewers, four ophthalmology residents of Ulsan University Hospital evaluated the fundus photographs and compared the results of evaluating fundus photographs with reference to AI results. A total of 180 fundus photographs were evaluated by extracting 20 images for each retinal disease. The 180 fundus photographs composed of eight types of retinal diseases and normal control group were randomly mixed, and each fundus photograph was read through a monitor for a sufficient time. At the second reading, the reading was performed while showing the result of the AI reading on the fundus photograph. A new AI model was trained by using the rest of the fundus photos as a training dataset, except for 180 photos, and we compared the results of the AI model’s diagnosis of 180 fundus photos with the results of residents. The accuracy of AI was 83.9%, which was lower than that of four ophthalmology residents. The accuracy of the results of diagnosis by four ophthalmology residents referring to the AI result slightly improved. However, residents showed reduced time spent by approximately 34% to 70% overall when they were notified of AI diagnosis results in advance (Table 6).

## 4. Discussion

Deep learning AI-based diagnostic tools are currently adopted by medical experts. In ophthalmology, AI algorithms extracting fundus images of retinal pathologies are potentially suitable for the development of image-based diagnostic systems. The early differential diagnosis of retinal diseases, such as dAMD, nAMD, DR, ERM, RRD, RP, MH, and RVO, is critical for appropriate treatment [21]. In this study, we conducted two-class diagnosis with AI tool between normal condition and retinal diseases (eight diseases) and showed a high accuracy (>99%) of VGG models. These results suggest that our AI-based tool is a promising approach for the preliminary diagnostic process. Furthermore, we developed an image-based AI diagnostic tool to detect and differentiate eight retinal diseases and normal control using fundus photograph.

Five-fold cross-validation revealed that the ResNet50 model had superior accuracy for nine-class classification compared with other models (VGG19 and Inception). Furthermore, the ResNet50 model with 128 nodes showed the highest accuracy (> 0.87). With this ResNet50 model, we compared the diagnostic performance of ophthalmology residents in the classification of nine retinal statuses (normal condition plus eight retinal diseases).

Four residents conducted differential diagnosis for nine classes and showed accuracy of 84.4%–91.7% without availability of preliminary outcome of the AI tool. However, they showed accuracy of 83.9%–92.2% when they could access the diagnostic outcome of the AI tool. Meanwhile, they shortened the time spent for diagnosis using AI tool’s reference data of up to 51 min (from 75 min to 24 min). Based on these results, the AI diagnostic model may be applicable to the preliminary process for an ophthalmologist’s differential diagnosis.

Some retinal diseases are difficult to differentially diagnose based on fundus photograph only. For example, in the case of DR and RVO, it may be difficult to distinguish through fundus photograph only if it is not typical. Even in the case of MH, accurate diagnosis may be difficult with fundus photograph only. In such diseases, additional patient information (medical history, medication, VA) and various types of retinal imaging examinations (OCT, FA/ICGA) are required for accurate diagnosis. However, it is difficult to demonstrate with an algorithm, and additional ophthalmic equipment is required. Therefore, to compensate for this shortcoming, we were able to increase the effectiveness of fundus diagnosis by showing the three high-priority diagnoses in order of % in the case of retinal diseases where differential diagnosis is difficult with fundus photograph only.

Some retinal diseases such as AMD and RP, which were also covered in this study, can be expressed in various forms by genetic changes related to RPE cells and photoreceptor cells [22,23]. From a future perspective, by expanding the area of application of the image-based AI tools to these diseases, it is also useful for differential diagnosis of retinal diseases in phenotype-genotype association studies, which take into account mutations in already known causative genes and biochemical pathways related.

Our study has several limitations. First, the age distributions among subject groups did not match because the age at which the disease occurs differs according to the disease and a sufficient number of suitable fundus photographs were not secured. Second, the fundus photos used in our study have two types of resolution, and to minimize the difference, it was converted to the same resolution of 512 × 512 pixels through a preprocessing step and then used for AI learning. Although the resolution was identically matched through the preprocessing, there is a possibility that this process did not completely eliminate the difference between the two types of pictures. Third, with the help of our AI tool, the reading time was shortened, but the improvement in accuracy was not as high as the reduction in time. We also think the most important thing is to improve the accuracy of the diagnosis. However, we have shown that this study can help in the differential diagnosis of various retinal diseases through AI deep learning.

## 5. Conclusions

Developing diagnostic tools for accurate diagnosis and prognostic prediction is required for the improvement of diagnostic accuracy and reduction of medical cost. Furthermore, AI-based platform and technology may resolve the low reliability of diagnostic imaging equipment in low-income countries. Therefore, we developed an image-based AI tool for differential diagnosis of retinal diseases using fundus photograph. These results suggest that our AI tool supports medical specialists to accurately diagnose retinal diseases in a cost-effective way.

## Figures and Tables

**Table 1:** Eyes (*n* = 549) were diagnosed with dAMD, nAMD, DR, ERM, RRD, RP, MH, or RVO, and fundus images were collected. Furthermore, 79 eyes from healthy subjects were collected as controls.

Disease		dAMD	nAMD	DR	ERM	RRD	RP	MH	RVO	Control
Fundus images (*n*)		58	79	95	99	80	50	49	39	79
Gender	Male	27	41	53	53	47	26	31	19	40
	Female	31	38	42	46	33	24	18	20	39
Age (years)		69.6 ± 8.0	69.1 ± 8.3	53.2 ± 10.4	63.6 ± 7.6	54.4 ± 14.6	53.4 ± 11.0	64.2 ± 8.9	67.5 ± 8.0	56.7 ± 7.3

Age (years) are presented as mean ± standard deviation. dAMD, dry age-related macular degeneration; nAMD, neovascular age-related macular degeneration; DR, diabetic retinopathy; ERM, epiretinal membrane; RRD, rhegmatogenous retinal detachment; RP, retinitis pigmentosa; MH, macular hole; RVO, retinal vein occlusion.

**Table 2:** The values of each argument used in ImageDataGenerator.

	Argument	Value
(1)	Width_shift_range	0.4
(2)	Height_shift_range	0.2
(3)	Rotation_range	90
(4)	Zoom_range	0.1
(5)	Horizontal_flip	True
	Vertical_flip	
(6)	Shear_range	30

**Table 3:** Comparison of outcomes of convolutional neural network models in two-class (normal vs. disease) diagnosis (accuracy).

Model	VGG19	Inception v3	ResNet50
Accuracy	99.12%	98.08%	97.85%

VGG19, Visual Geometry Group with 19 layers; ResNet50, Deep Residual Learning for Image Recognition with 50 layers.

**Table 4:** Accuracy results obtained using 5-fold cross-validation.

Dense Layer	VGG19	Inception v3	ResNet50
128 nodes	0.8200 ± 0.0282	0.8340 ± 0.0364	0.8742 ± 0.0349
256 nodes	0.8135 ± 0.0315	0.8212 ± 0.0444	0.8646 ± 0.0205
128 nodes + 128 nodes	0.8168 ± 0.0243	0.8360 ± 0.0115	0.8694 ± 0.0338
256 nodes + 256 nodes	0.8026 ± 0.0365	0.8483 ± 0.0381	0.8452 ± 0.0351

The data are shown as mean ± standard deviation. VGG19, Visual Geometry Group with 19 layers; ResNet50, Deep Residual Learning for Image Recognition with 50 layers.

**Table 5:** Cross-validation results of classification performance evaluation index for nine-class diagnosis (accuracy, sensitivity, specificity, PPV, NPV).

Model	Accuracy	Class	Sensitivity	Specificity	PPV	NPV
ResNet50 with 128 nodes	87.42%	dAMD	0.8190	0.9844	0.8439	0.9770
		DR	0.9262	0.9833	0.9052	0.9868
		ERM	0.9252	0.9830	0.9089	0.9850
		MH	0.8192	0.7960	0.7556	0.9861
		Normal	0.8830	0.9873	0.9092	0.9800
		RP	0.9085	0.9966	0.9600	0.9914
		RRD	0.8143	0.9870	0.9125	0.9671
		RVO	0.8514	0.9916	0.8750	0.9882
		wAMD	0.9708	0.9667	0.7600	0.9964

NPV, negative predictive value; PPV, positive predictive value; ResNet50, Deep Residual Learning for Image Recognition with 50 layers.

**Table 6:** Comparison of diagnostic results before and after AI reference. A total of 180 fundus photos were evaluated by extracting 20 images for each retinal disease.

	AI Results	Ophthalmology Residents’ Results							
		Before Referring to AI Results				After Referring to AI Results			
		1	2	3	4	1	2	3	4
Wrong count	29	15	21	28	27	14	17	29	23
Accuracy (%)	83.9	91.7	88.3	84.4	85	92.2	90.6	83.9	87.2
Time (min)		50	70	75	32	15	25	24	25

## Data Availability

The data generated during and/or analyzed the current study are available from the corresponding author on reasonable request.
